# Field Theory Approaches to Relativistic Hydrodynamics

**DOI:** 10.3390/e24121790

**Published:** 2022-12-07

**Authors:** Nahuel Mirón Granese, Alejandra Kandus, Esteban Calzetta

**Affiliations:** 1 Consejo Nacional de Investigaciones Científicas y Técnicas (CONICET), Rivadavia 1917, Ciudad de Buenos Aires CP 1033, Argentina; 2 Facultad de Ciencias Astronómicas y Geofísicas, Universidad Nacional de La Plata, Paseo del Bosque, La Plata CP 1900, Argentina; 3 Departamento de Física, Facultad de Ciencias Exactas y Naturales, Universidad de Buenos Aires, Ciudad Universitaria, Ciudad de Buenos Aires CP 1428, Argentina; 4 LATO-DCET, Universidade Estadual de Santa Cruz, Rodov. J. Amado km 16, Salobrinho, Ilhéus 45662-900, BA, Brazil; 5 Instituto de Física de Buenos Aires (IFIBA), y CONICET—Universidad de Buenos Aires, Ciudad de Buenos Aires CP 1428, Argentina

**Keywords:** relativistic hydrodynamics, quantum field theory, hydrodynamic fluctuations

## Abstract

Just as non-relativistic fluids, oftentimes we find relativistic fluids in situations where random fluctuations cannot be ignored, with thermal and turbulent fluctuations being the most relevant examples. Because of the theory’s inherent nonlinearity, fluctuations induce deep and complex changes in the dynamics of the system. The Martin–Siggia–Rose technique is a powerful tool that allows us to translate the original hydrodynamic problem into a quantum field theory one, thus taking advantage of the progress in the treatment of quantum fields out of equilibrium. To demonstrate this technique, we shall consider the thermal fluctuations of the spin two modes of a relativistic fluid, in a theory where hydrodynamics is derived by taking moments of the Boltzmann equation under the relaxation time approximation.

## 1. Introduction

The success of hydrodynamics in the description of relativistic heavy ion collisions [1,2,3] has brought to the fore the problem of the very early stages of the process, as hydrodynamic behavior appeared on time scales that were not longer than the expected relaxation times [4,5,6,7,8,9,10,11]. In a parallel development, the possibility of relativistic viscous fluids playing an important role in the evolution of electromagnetic and gravitational background fields in cosmological and astrophysical settings [12,13,14,15,16,17,18,19] has similarly brought attention to regimes where the relaxation time is not the shortest time scale in the problem.

In this regime, not only is the fluid strongly out of equilibrium, but also its fluctuations are not negligible. To mention two important instances of this phenomenon, these fluctuations may be of thermal origin, or else due to the nonlinear amplification of external influences leading to turbulence [20,21,22,23,24,25,26].

Field theory methods, through the so-called Martin–Siggia–Rose (MSR) approach [27,28,29,30], have proven to be powerful tools in the analysis of fluctuating fluids. The method is based on the construction of a generating functional for the correlation functions of the fluid [31,32,33,34,35,36,37,38]. This functional has the same form as the generating functional for a non-equilibrium quantum field [39], and this brings to bear the substantial tools available for the study of these systems, particularly functional methods based on the one- or two-particle irreducible effective action. The first choice is relevant when we are interested in the mean values of the hydrodynamic variables, while the second is superior when the goal is the description of correlations, which is the task at hand.

In this paper, we shall present the MSR approach to fluctuating relativistic viscous fluids. Hydrodynamics is conceived as an effective theory describing the long-lived modes of a more fundamental description, which is usually either a field theory [39] (including conformal field theories, which may be studied though holographic methods [40]) or else a kinetic theory [41]. For concreteness, we shall assume the latter. We start from a Boltzmann equation (as we shall discuss below, for the kind of problems we have in mind it is enough to work within the relaxation time approximation for the collision term [42,43,44,45,46]) and derive hydrodynamics by taking moments of this equation [47,48] in a manner to be fully described below.

As mentioned above, in applications, the emphasis is on the interaction of the fluid with electromagnetic and gravitational fields; therefore, the main interest is on the spin 1 and 2 modes of the fluid. We have treated electromagnetic interactions elsewhere (including the possibility of the fluid amplifying a seed field through the Weibel instability [49,50]); in this paper, we shall focus on spin 2 modes.

The existence of non-hydrodynamic tensor modes in a relativistic fluid is a generic prediction of kinetic theory [51,52,53]. To capture them, we must recur to a particular parameterization of the one-particle distribution function, including explicitly a second-order tensor as an independent hydrodynamic variable; as we shall show below, to provide this mode with a finite propagation speed, we must include a third-order tensor as well [53,54,55]. As a test case for the formalism, we shall formulate a minimal model including a dynamical tensor mode and shall study how nonlinearities modify the spectrum of the thermal fluctuation of this mode. Concretely, we shall show that the equal time thermal spectrum is flat at long wavelengths (as it is in the linear theory) but becomes a power law at short ones, resembling the spectra characteristic of relativistic turbulence [26].

This paper is organized as follows. In the next section, we formulate the MSR approach in the language of the two-particle irreducible effective action (2PIEA). We conclude by stating the concrete form of the dressed correlations, to be computed in the following.

We then switch to a presentation of stochastic hydrodynamics, as derived from the moments of a stochastic kinetic equation [56,57,58,59,60,61] after a parameterization of the one-particle distribution function, which includes, besides temperature and four velocity, the tensor hydrodynamic variables required to capture the spin 2 non-hydrodynamic mode of the fluid [53]. We consider a massless relativistic gas; so, we shall not include chemical potential among the hydrodynamic variables.

Finally, we deploy the MSR tools to compute the dressed correlations of the tensor modes to one-loop accuracy. This requires computing corrections to both the causal propagator (the so-called self energy) and to the noise correlation (the so-called noise kernel); together, they determine the tensor mode correlation. For easier comparison with the correlation of the linear theory, we compute the equal time correlation, thus obtaining the dressed spectrum of spin two fluctuations.

We conclude with some brief final remarks. The details of the calculation of the relevant Feynman graphs are given in Appendix A.

## 2. The Martin–Siggia–Rose Approach

### 2.1. From Stochastic to Quantum Fields

Let us begin by reviewing how one can translate a stochastic field theory into a quantum one [27,28,29,30]. One has a theory of fields $X^{\alpha }$ (the α index accounts for space-time, Lorentz and species indexes) obeying nonlinear stochastic equations of motion

(1) $$P^{a}=D_{\beta }^{a}X^{\beta }+\Lambda_{\beta \gamma }^{a}X^{\beta }X^{\gamma }+\ldots =F^{a}$$

The $F^{a}$ are assumed to be a Gaussian noise with zero mean and self correlation

(2) $$\langle F^{a}F^{b}\rangle =\Phi^{ab}$$

The fields $X^{\alpha }$ become stochastic themselves, with a probability density functional

(3) $$X\left[X^{\alpha }\right]=\int \;DF^{b}\;F\left[F^{b}\right]\delta \left[X^{\alpha }-X^{\alpha }\left[F^{b}\right]\right]$$

where $X^{\alpha }\left[F^{b}\right]$ is the solution to Equation (1) for a given realization $F^{b}$ of the noise, and

(4) $$F\left[F^{b}\right]=C\;\exp\left\{-\frac{1}{2}F^{b}\Phi_{bc}^{-1}F^{c}\right\}$$

is the probability density functional for the noise.

For simplicity, we assume that the $X^{\alpha }$ fields do not develop a nonzero expectation value. Then, the interest lies on the self correlation

(5) $$G_{1}^{\alpha \beta }=\langle X^{\alpha }X^{\beta }\rangle$$

$G_{1}^{\alpha \beta }$ may be derived from a generating functional

(6) $$G_{1}^{\alpha \beta }=2{\left.\frac{\delta W\left[K_{\beta \gamma }\right]}{\delta K_{\alpha \beta }}\right|}_{K_{\alpha \beta }=0}$$

(7) $$\begin{array}{ccc} e^{iW\left[K_{\beta \gamma }\right]} & = & \int \;DX^{\alpha }\;X\left[X^{\alpha }\right]\;\exp\left\{\frac{i}{2}X^{\alpha }K_{\alpha \beta }X^{\beta }\right\}\\ & = & \int \;DX^{\alpha }DF^{b}\;F\left[F^{b}\right]\delta \left[X^{\alpha }-X^{\alpha }\left[F^{b}\right]\right]\;\exp\left\{\frac{i}{2}X^{\alpha }K_{\alpha \beta }X^{\beta }\right\} \end{array}$$

We now proceed as follows. First, we use the identity

(8) $$\delta \left[X^{\alpha }-X^{\alpha }\left[F^{\beta }\right]\right]=Det\left[\frac{\delta P^{a}}{\delta X^{\beta }}\right]\delta \left[P^{a}-F^{a}\right]$$

The δ-functions may be added to the exponent by introducing auxiliary fields $Y_{a}$ and to the determinant by adding ghost fields. It can be shown that ghosts play no role in the discussion below; so, we shall simply assume that the determinant is a constant [62]. So, we obtain

(9) $$e^{iW\left[K_{\beta \gamma }\right]}=\int \;DY_{a}DX^{\alpha }DF^{b}\;F\left[F^{b}\right]\;\exp\left\{iY_{a}\left(P^{a}-F^{a}\right)+\frac{i}{2}X^{\alpha }K_{\alpha \beta }X^{\beta }\right\}$$

Finally, we integrate over the noises $F^{a}$ to obtain

(10) $$e^{iW\left[K_{\beta \gamma }\right]}=\int \;DY_{a}DX^{\alpha }\;\exp\left\{iY_{a}P^{a}-\frac{1}{2}Y_{a}\Phi^{ab}Y_{b}+\frac{i}{2}X^{\alpha }K_{\alpha \beta }X^{\beta }\right\}$$

At this point, it is convenient to consider $X^{A}=\left(X^{\alpha },Y_{a}\right)$ as a single field and add sources as necessary so that we obtain a generating functional for the correlations $G^{AB}=\langle X^{a}X^{b}\rangle$, namely,

(11) $$e^{iW\left[K_{\beta \gamma }\right]}=\int \;DX^{A}\;\exp\left\{iS\left[X^{A}\right]+\frac{i}{2}X^{A}K_{AB}X^{b}\right\}$$

where

(12) $$S\left[X^{A}\right]=Y_{a}P^{a}+\frac{i}{2}Y_{a}\Phi^{ab}Y_{b}$$

The “doubling of degrees of freedom” gives this the structure of the classical action for a quantum field defined on a *closed time-path* [39]. By performing a Lagrange transform with respect to $K_{AB}$, we obtain the *two-particle irreducible effective action* (2PIEA)

(13) $$\Gamma \left[G^{AB}\right]={\left.W\left[K_{AB}\right]-\frac{1}{2}K_{AB}G^{AB}\right|}_{W_{,K}=G/2}$$

The variation of the 2PIEA yields the Schwinger–Dyson equations

(14) $$\frac{\delta \Gamma }{\delta G^{AB}}=0$$

which are the most efficient way to find the correlations.

The effective action approach has points in common with the non-equilibrium generating functional introduced by Zubarev [63,64,65,66]; however, the goal here is not to compute the correlation functions directly from a generating functional, but rather, the equations of motion thereof, which are similar to the Schwinger–Dyson equations from field theory.

We should emphasize that the reason to appeal to functional methods is to make efficient use of the information already encoded in the equations of motion. In principle, one could use field theory methods without introducing path integrals, as was conducted by Wyld [67]. However, the path integral formulation makes it easier to implement powerful methods such as the functional renormalization group [68,69,70], which we aim to discuss in future publications. For further discussion of path integral methods, see [71].

### 2.2. Mining the 2PIEA

The 2PIEA has the structure [39]

(15) $$\Gamma \left[G^{AB}\right]=\frac{1}{2}{\left.\frac{\delta^{2}S}{\delta X^{A}\delta X^{B}}\right|}_{X^{A}=0}G^{AB}-\frac{i}{2}\ln\left[Det\;G^{AB}\right]+\Gamma_{Q}\left[G^{AB}\right]$$

where $\Gamma_{Q}$ is the sum of all two-particle irreducible vacuum graphs with the full propagator $G^{AB}$ and vertices derived from the interaction action

(16) $$S_{Q}=Y_{a}\left\{\Lambda_{\beta \gamma }^{a}X^{\beta }X^{\gamma }+\ldots \right\}$$

therefore, the Schwinger–Dyson equations are

(17) $${\left.\frac{\delta^{2}S}{\delta X^{A}\delta X^{B}}\right|}_{X^{A}=0}-iG_{AB}^{-1}+2\frac{\delta \Gamma_{Q}}{\delta G^{AB}}=0$$

The analysis of these equations is greatly simplified by the observation that

(18) $$G_{ab}=\langle Y_{a}Y_{b}\rangle =0$$

Then, the obvious identity

(19) $$\left(\begin{array}{cc} G_{\alpha \beta }^{-1} & G_{\alpha }^{-1b}\\ G_{\;\;\;\;\;\beta }^{-1a} & G^{-1ab} \end{array}\right)\left(\begin{array}{cc} \langle X^{\beta }X^{\gamma }\rangle & \langle X^{\beta }Y_{c}\rangle \\ \langle Y_{b}X^{\gamma }\rangle & 0 \end{array}\right)=\left(\begin{array}{cc} \delta_{\alpha }^{\gamma } & 0\\ 0 & \delta_{c}^{a} \end{array}\right)$$

shows that $\langle X^{\beta }Y_{c}\rangle$ is invertible, since

(20) $$G_{\;\;\;\;\;\beta }^{-1a}\langle X^{\beta }Y_{c}\rangle =\delta_{c}^{a}$$

and then, the further equation $G_{\alpha \beta }^{-1}\langle X^{\beta }Y_{c}\rangle =0$ shows that $G_{\alpha \beta }^{-1}=0$. So, there are only two families of nontrivial Schwinger–Dyson equations, Equation (20) and

(21) $$G_{\;\;\;\;\;\beta }^{-1a}\langle X^{\beta }X^{\gamma }\rangle +G^{-1ab}\langle Y_{b}X^{\gamma }\rangle =0$$

or else, reading the inverse propagators from the Schwinger–Dyson equations,

(22) $$\begin{array}{ccc} \left(-i\right)\left[D_{\beta }^{a}+2\frac{\delta \Gamma_{Q}}{\delta \langle Y_{a}X^{\beta }\rangle }\right]\langle X^{\beta }Y_{c}\rangle & = & \delta_{c}^{a}\\ \left(-i\right)\left[D_{\beta }^{a}+2\frac{\delta \Gamma_{Q}}{\delta \langle Y_{a}X^{\beta }\rangle }\right]\langle X^{\beta }X^{\gamma }\rangle +\left[\Phi^{ab}-2i\frac{\delta \Gamma_{Q}}{\delta \langle Y_{a}Y_{b}\rangle }\right]\langle Y_{b}X^{\gamma }\rangle & = & 0 \end{array}$$

The propagator

(23) $$\langle X^{\beta }Y_{c}\rangle =i\langle \frac{\delta X^{\beta }}{\delta F^{c}}\rangle$$

is causal. It is the retarded propagator of the theory. By symmetry, $\langle Y_{b}X^{\gamma }\rangle$ is the advanced propagator. The propagator $\langle X^{\beta }X^{\gamma }\rangle =G_{1}^{\beta \gamma }$ is the physical correlation function of the theory. We now see that we could derive $G_{1}^{\beta \gamma }$ from a stochastic equation

(24) $$\left[D_{\beta }^{a}+\Sigma_{\beta }^{a}\right]X^{\beta }=F_{dressed}^{a}$$

where the self-energy

(25) $$\Sigma_{\beta }^{a}=2\frac{\delta \Gamma_{Q}}{\delta \langle Y_{a}X^{\beta }\rangle }$$

and the dressed noise has a self-correlation

(26) $$\langle F_{dressed}^{a}F_{dressed}^{b}\rangle \equiv N^{ab}=\Phi^{ab}-2i\frac{\delta \Gamma_{Q}}{\delta \langle Y_{a}Y_{b}\rangle }$$

where $N^{ab}$ is the so-called *noise kernel*.

### 2.3. The Lowest-Order Correlation

To make the analysis above more concrete, we shall compute the lowest-order correction to the self-energy and to the noise kernel. Keeping only the quadratic terms in the original stochastic equation, and assuming that $\Lambda_{\beta \gamma }^{a}$ is symmetric on $\left(\beta ,\gamma \right)$, the lowest-order contribution to $\Gamma_{Q}$ is

(27) $$\begin{array}{ccc} \Gamma_{Q} & = & \frac{i}{2}\Lambda_{\beta \gamma }^{a}\Lambda_{\beta^{\prime }\gamma^{\prime }}^{a^{\prime }}{\langle \left(Y_{a}X^{\beta }X^{\gamma }\right)\left(Y_{a^{\prime }}X^{\beta^{\prime }}X^{\gamma^{\prime }}\right)\rangle }_{2PI}\\ & = & 2i\Lambda_{\beta \gamma }^{a}\Lambda_{\beta^{\prime }\gamma^{\prime }}^{a^{\prime }}\langle Y_{a}X^{\beta^{\prime }}\rangle \langle X^{\beta }Y_{a^{\prime }}\rangle \langle X^{\gamma }X^{\gamma^{\prime }}\rangle \\ & + & i\Lambda_{\beta \gamma }^{a}\Lambda_{\beta^{\prime }\gamma^{\prime }}^{a^{\prime }}\langle Y_{a}Y_{a^{\prime }}\rangle \langle X^{\beta }X^{\beta^{\prime }}\rangle \langle X^{\gamma }X^{\gamma^{\prime }}\rangle \end{array}$$

and so

(28) $$\begin{array}{ccc} \Sigma_{\beta }^{a} & = & 4i\Lambda_{\beta^{\prime }\gamma }^{a}\Lambda_{\beta \gamma^{\prime }}^{a^{\prime }}\langle X^{\beta^{\prime }}Y_{a^{\prime }}\rangle \langle X^{\gamma }X^{\gamma^{\prime }}\rangle \\ N^{ab} & = & \Phi^{ab}+2\Lambda_{\beta \gamma }^{a}\Lambda_{\beta^{\prime }\gamma^{\prime }}^{b}\langle X^{\beta }X^{\beta^{\prime }}\rangle \langle X^{\gamma }X^{\gamma^{\prime }}\rangle \end{array}$$

On the right-hand side of (28), we may use the lowest-order propagators

(29) $$\begin{array}{ccc} \langle X^{\beta^{\prime }}Y_{a^{\prime }}\rangle & = & i{\left[D^{-1}\right]}_{a^{\prime }}^{\beta^{\prime }}\\ \langle X^{\beta }X^{\beta^{\prime }}\rangle & = & \left(-1\right){\left[D^{-1}\right]}_{a}^{\beta }\;\Phi^{aa^{\prime }}\;{\left[D^{-1}\right]}_{a^{\prime }}^{\beta^{\prime }} \end{array}$$

Finally, the dressed propagators read

(30) $$\begin{array}{c} {\langle X^{\beta^{\prime }}Y_{a^{\prime }}\rangle }_{dressed}=i{\left[D+\Sigma \right]}^{-1}{}_{a^{\prime }}^{\beta^{\prime }} \end{array}$$

(31) $$\begin{array}{c} {\langle X^{\beta }X^{\beta^{\prime }}\rangle }_{dressed}=\left(-1\right){\left[D+\Sigma \right]}^{-1}{}_{a}^{\beta }\;N^{aa^{\prime }}\;{\left[D+\Sigma \right]}^{-1}{}_{a^{\prime }}^{\beta^{\prime }} \end{array}$$

(32) $$\begin{array}{c} ={\langle X^{\beta }Y_{a}\rangle }_{dressed}\;N^{aa^{\prime }}\;{\langle Y_{a^{\prime }}X^{\beta^{\prime }}\rangle }_{dressed} \end{array}$$

## 3. From Stochastic Kinetic Theory to Stochastic Hydrodynamics

### 3.1. Relativistic Kinetic Theory

Hydrodynamics is usually conceived as an effective theory that captures the dynamics of the long-lived modes of a more fundamental description [72,73]—in practice, either field theory (including holographic models) or kinetic theory. In this article, we shall take the latter viewpoint; so, it is convenient to start with a brief comment on relativistic kinetic theory [41].

We shall consider the kinetic theory of massless, neutral particles. They are described by the one-particle distribution function $f\left(x^{\mu },p_{\nu }\right)$, where $p^{2}=0$ and $p^{0}\geq 0$. The energy-momentum tensor is

(33) $$T^{\mu \nu }=\int \;Dp\;p^{\mu }p^{\nu }f$$

and the entropy is

(34) $$S^{\mu }=\int \;Dp\;p^{\mu }f\left[1-\ln f\right]$$

Here,

(35) $$Dp=\frac{2d^{4}p_{\nu }}{{\left(2\pi \right)}^{3}}\delta \left(-p^{2}\right)\theta \left(p^{0}\right)$$

is the invariant momentum space measure. Once $T^{\mu \nu }$ is given, we define the fluid velocity $u^{\mu }$, temperature *T* and inverse temperature vector $\beta^{\mu }=u^{\mu }/T$ from the Landau–Lifshitz prescription $T^{\mu \nu }u_{\nu }=-\rho u^{\mu }$, where $\rho =3T^{4}/\pi^{2}$ is the energy density and $u^{2}=-1$. The equation of motion is Boltzmann’s

(36) $$p^{\mu }\frac{\partial f}{\partial X^{\mu }}=I_{col}.$$

The collision integral is restricted by energy-momentum conservation

(37) $$\int \;Dp\;p^{\mu }\;I_{col}=0$$

and the *H* theorem

(38) $$\int \;Dp\;\ln f\;I_{col}\leq 0$$

for *any* solution of Equation (36); this enforces the Second Law $S_{,\mu }^{\mu }=\sigma \geq 0$.

We shall assume that the collision integral expanded to linear order around an equilibrium solution defines a symmetric operator on the space of linear perturbations to the one-particle distribution function. Then, because of (37), this operator must have four null eigenvectors associated to the momenta $p^{\mu }$. Since we are considering a massless gas, we do not enforce particle number conservation. We assume these are the only null eigenvectors—they are the hydrodynamic modes.

The rest of the eigenvectors to the collision operator have negative eigenvalues. We shall consider “hard” collision terms, where there is a finite gap between zero and the first nonzero eigenvalue, as opposed to “soft” collision terms where there is a continuous spectrum stretching away from zero [74,75,76]. For the present discussion, a hard collision term may be accurately approximated by an Anderson–Witting or relaxation time approximation collision term [42,43,77,78,79,80]

(39) $$I_{col}=\frac{-1}{\tau }\left(-u_{\sigma }p^{\sigma }\right)\left[f-f_{0}\right]$$

where $f_{0}$ is an equilibrium solution. Momentum conservation requires $f_{0}$ to be the equilibrium distribution built from the inverse temperature vector derived from *f*, and $u^{\mu }$ to be the corresponding Landau–Lifshitz velocity.

Of course, this is not the only possible linear approximation to the collision term, just one of the best known, together with Marle’s [81]. Over time, other proposals have been advanced, with the goal of allowing for a momentum dependence of the relaxation time [45,46,73,82] and/or to account for both elastic and inelastic collisions [83].

The non-null eigenvectors of the collision operator are associated to the non-hydrodynamic modes. The existence of a spin 2 non-hydrodynamic mode is a generic prediction of kinetic theory [53]. For example, if the velocity lies in the *z* direction, then a perturbation proportional to $p_{x}p_{y}$ would contribute to the spin 2 part of the energy momentum tensor. This perturbation must have some nontrivial expansion in terms of collision operator eigenvectors, since it is orthogonal to the hydrodynamic modes.

Most importantly, tensor modes mediate the interaction between the fluid and gravitational waves, both in cosmological scenarios such as the post-inflationary Universe [13,18,19] or the phase transitions era [16] and in astrophysical scenarios such as rotating compact objects [14,17] and merging neutron stars [12]. Therefore, we must understand the dynamics of those modes to correctly describe these phenomena.

### 3.2. The Moments Approach to Hydrodynamics

To obtain hydrodynamics from kinetic theory, we begin by expanding lnf in a set of functions $f_{\alpha }\left(x^{\mu },p_{\nu }\right)$ [84]

(40) $$\ln f=\sum_{\alpha }X^{\alpha }f_{\alpha }\left(x^{\mu },p_{\nu }\right)$$

It is customary to choose $\beta^{\mu }$ as one of the $X^{\alpha }$, with $p^{\mu }$ as the corresponding $f_{\alpha }$. Hydrodynamics follows from the truncation of this development to a (hopefully) few terms; the $X^{\alpha }$ then become the hydrodynamic variables. To obtain the hydrodynamic equations, we take moments of the Boltzmann equation with suitable functions $R^{a}$

(41) $$\int \;Dp\;R^{a}\left(x^{\nu },p_{\nu }\right)\left[p^{\mu }\frac{\partial f}{\partial x^{\mu }}-I_{col}\right]=0$$

The problem is that the truncated *f* is not a solution of the Boltzmann equation and, so, we cannot appeal to the *H* theorem to enforce the Second Law. The way out is to choose the $R^{a}$ as the $f_{\alpha }$ themselves. In particular, energy-momentum conservation $T_{,\nu }^{\mu \nu }=0$ becomes one the equations of motion.

It is clear that this scheme is still too general; to proceed, we must consider a particular realization. In this work, we shall restrict ourselves to

(42) $$\ln f=\beta_{\mu }p^{\nu }+X_{\mu \nu }\frac{p^{\mu }p^{\nu }}{\left(-u_{\sigma }p^{\sigma }\right)}+X_{\mu \nu \rho }\frac{p^{\mu }p^{\nu }p^{\rho }}{{\left(-u_{\sigma }p^{\sigma }\right)}^{2}}$$

It is assumed that the $X_{\mu \nu }$ and $X_{\mu \nu \rho }$ tensors are totally symmetric, transverse with respect to $u^{\mu }$ and traceless on any two indexes. The $X^{\mu \nu }$ field captures the tensor mode, which is our main concern; the $X^{\mu \nu \rho }$ field is then necessary to obtain nontrivial dynamics for those modes [53,55]. The model where $X^{\mu \nu \rho }$ is not included has been analyzed in [85].

The powers of $-u_{\sigma }p^{\sigma }$ in the denominators are included to avoid the non-equilibrium terms dominating the equilibrium one. If these powers are not included, the theory becomes a divergence-type model [86,87,88,89,90,91] but the momentum integrals become divergent and must be renormalized [92]. In any case, the application of these models to Bjorken and Gubser flows, where exact solutions to kinetic theory are available, and to relativistic shock waves, shows that including the denominators in (42) greatly improves the concordance with the exact results [54,84].

The corresponding equations of motion are

(43) $$\begin{array}{ccc} S_{\mu^{\prime }\nu^{\prime }}^{\mu \nu }\int \;Dp\;\frac{p^{\mu^{\prime }}p^{\nu^{\prime }}}{\left(-u_{\sigma }p^{\sigma }\right)}\left[p^{\lambda }\frac{\partial f}{\partial x^{\lambda }}-I_{col}\right] & = & 0\\ S_{\mu^{\prime }\nu^{\prime }\rho^{\prime }}^{\mu \nu \rho }\int \;Dp\;\frac{p^{\mu^{\prime }}p^{\nu^{\prime }}p^{\rho^{\prime }}}{{\left(-u_{\sigma }p^{\sigma }\right)}^{2}}\left[p^{\lambda }\frac{\partial f}{\partial x^{\lambda }}-I_{col}\right] & = & 0 \end{array}$$

where $S_{\mu^{\prime }\nu^{\prime }}^{\mu \nu }$ and $S_{\mu^{\prime }\nu^{\prime }\rho^{\prime }}^{\mu \nu \rho }$ are projectors over the space of totally symmetric, transverse with respect to $u^{\mu }$ and traceless tensors. They can be built from the projector $\Delta^{\mu \nu }=\eta^{\mu \nu }+u^{\mu }u^{\nu }$.

With an integration by parts, we may write

(44) $$\begin{array}{ccc} S_{\mu^{\prime }\nu^{\prime }}^{\mu \nu }\left[A_{,\lambda }^{\mu^{\prime }\nu^{\prime }\lambda }-K^{\mu^{\prime }\nu^{\prime }\sigma \lambda }u_{\sigma ,\lambda }-I^{\mu^{\prime }\nu^{\prime }}\right] & = & 0\\ S_{\mu^{\prime }\nu^{\prime }\rho^{\prime }}^{\mu \nu \rho }\left[A_{,\lambda }^{\mu^{\prime }\nu^{\prime }\rho^{\prime }\lambda }-2K^{\mu^{\prime }\nu^{\prime }\rho^{\prime }\sigma \lambda }u_{\sigma ,\lambda }-I^{\mu^{\prime }\nu^{\prime }\rho^{\prime }}\right] & = & 0 \end{array}$$

where

(45) $$\begin{array}{ccc} A^{\mu^{\prime }\nu^{\prime }\lambda } & = & \int \;Dp\;\frac{p^{\mu^{\prime }}p^{\nu^{\prime }}}{\left(-u_{\sigma }p^{\sigma }\right)}p^{\lambda }f\\ K^{\mu^{\prime }\nu^{\prime }\sigma \lambda } & = & A^{\mu^{\prime }\nu^{\prime }\sigma \lambda }=\int \;Dp\;\frac{p^{\mu^{\prime }}p^{\nu^{\prime }}}{{\left(-u_{\sigma^{\prime }}p^{\sigma^{\prime }}\right)}^{2}}p^{\lambda }p^{\sigma }f\\ I^{\mu^{\prime }\nu^{\prime }} & = & \int \;Dp\;\frac{p^{\mu^{\prime }}p^{\nu^{\prime }}}{\left(-u_{\sigma }p^{\sigma }\right)}I_{col}\\ K^{\mu^{\prime }\nu^{\prime }\rho^{\prime }\sigma \lambda } & = & \int \;Dp\;\frac{p^{\mu^{\prime }}p^{\nu^{\prime }}p^{\rho^{\prime }}}{{\left(-u_{\sigma^{\prime }}p^{\sigma^{\prime }}\right)}^{3}}p^{\lambda }p^{\sigma }f\\ I^{\mu^{\prime }\nu^{\prime }\rho^{\prime }} & = & \int \;Dp\;\frac{p^{\mu^{\prime }}p^{\nu^{\prime }}p^{\rho^{\prime }}}{{\left(-u_{\sigma }p^{\sigma }\right)}^{2}}I_{col} \end{array}$$

With the Anderson–Witting collision term (39), we obtain

(46) $$\begin{array}{ccc} I^{\mu^{\prime }\nu^{\prime }} & = & \frac{-1}{\tau }\left[T^{\mu^{\prime }\nu^{\prime }}-T_{0}^{\mu^{\prime }\nu^{\prime }}\right]\\ I^{\mu^{\prime }\nu^{\prime }\rho^{\prime }} & = & \frac{-1}{\tau }\left[A^{\mu^{\prime }\nu^{\prime }\rho^{\prime }}-A_{0}^{\mu^{\prime }\nu^{\prime }\rho^{\prime }}\right] \end{array}$$

The second terms, where *f* is replaced by $f_{0}$, will not survive the projector operators and may be discarded.

### 3.3. Stochastic Hydrodynamics

The above framework is incomplete in that it does not account for thermal fluctuations. We may fix that by adding a noise term to the Boltzmann equation as derived from fluctuation–dissipation considerations [56,57,58,59,60,61].

Let us first consider the fluctuation–dissipation theorem in an abstract setting. Suppose a theory with variables $x^{\alpha }$ whose probability density, in equilibrium, takes the form

(47) $$f\left(x^{\alpha }\right)=e^{\Phi \left(x^{\alpha }\right)}$$

For example, if the system is in thermal equilibrium, then the potential Φ is −F/T, where *F* is the free energy F=E−TS.

Equation (47) implies that the system is not just sitting at the equilibrium state, which is the maximum of the potential—which we identify as $x^{\alpha }=0$—but fluctuates around it, in a way that is prescribed by the classical equipartition theorem

(48) $$\langle x^{\alpha }J_{\beta }\rangle =\delta_{\beta }^{\alpha }$$

where

(49) $$J_{\beta }=-\frac{\partial \Phi }{\partial x^{\beta }}$$

is the so-called thermodynamic force.

The dynamics of the system has a deterministic and a random component

(50) $${\dot{x}}^{\alpha }=F_{det}^{\alpha }+\zeta^{\alpha }$$

The deterministic part drags the system towards $x^{\alpha }=0$; for linear deviations from equilibrium, it may be parameterized as

(51) $$F_{det}^{\alpha }=-\gamma^{\alpha \beta }J_{\beta }$$

where the matrix $\gamma^{\alpha \beta }$ is positive definite. Then, the fluctuation–dissipation theorem states that

(52) $$\langle \zeta^{\alpha }\zeta^{\beta }\rangle =\gamma^{\alpha \beta }+\gamma^{\beta \alpha }$$

Let us apply this general scheme to kinetic theory. The relevant thermodynamic potential is the Massieu function

(53) $$\Phi =-\int \;d^{3}x\;U_{\mu }\Phi^{\mu }$$

where $U^{\mu }$ is the equilibrium four velocity, namely, the velocity of an observer at rest with respect to the thermal bath (which we take as $\left(1,0,0,0\right)$) [93]

(54) $$\Phi^{\mu }=S^{\mu }+\beta_{0\nu }T^{\mu \nu }=-\int \;Dp\;p^{\mu }f\left[\ln f-1-\beta_{0\nu }p^{\nu }\right]$$

Upon a perturbation $f=f_{0}+\delta f$, we have [94]

(55) $$\begin{array}{ccc} \Phi^{\mu } & = & -\int \;Dp\;p^{\mu }\left(f_{0}+\delta_{f}\right)\left[\frac{\delta f}{f_{0}}-\frac{1}{2}{\left(\frac{\delta f}{f_{0}}\right)}^{2}-1\right]\\ & = & \Phi_{0}^{\mu }-\frac{1}{2}\int \;Dp\;p^{\mu }\frac{\delta f^{2}}{f_{0}} \end{array}$$

The thermodynamic force is derived from the variational derivative of the global Massieu function (53).

(56) $$J\left(\left(t,\vec{x}\right),\vec{p}\right)=-\frac{\delta \Phi }{\delta f\left(\left(t,\vec{x}\right),\vec{p}\right)}=\frac{1}{{\left(2\pi \right)}^{3}}\frac{\delta f}{f_{0}}\left(\left(t,\vec{x}\right),\vec{p}\right)$$

To write the Anderson–Witting collision term (39) to the required order, we need to compute the corrections to $f_{0}$. We start from

(57) $$T^{\mu \nu }=T_{0}^{\mu \nu }+\delta T^{\mu \nu }$$

so, writing $u^{\mu }=U^{\mu }+\delta u^{\mu }$, $T=T_{0}+\delta T$ and $U_{\mu }\delta u^{\mu }=0$ we have

(58) $$\left[T_{0}^{\mu \nu }+\delta T^{\mu \nu }\right]\left[U_{\nu }+\delta u_{\nu }\right]=-\left[\rho_{0}+\delta \rho \right]\left[U^{\mu }+\delta u^{\mu }\right]$$

To first order

(59) $$T_{0}^{\mu \nu }\delta u_{\nu }+\delta T^{\mu \nu }U_{\nu }=-\rho_{0}\delta u^{\mu }-\delta \rho U^{\mu }$$

Therefore,

(60) $$\begin{array}{ccc} \delta \rho & = & \delta T^{\mu \nu }U_{\mu }U_{\nu }\\ \delta u^{\mu } & = & -\frac{\Delta_{\rho }^{\mu }\delta T^{\rho \nu }U_{\nu }}{\rho_{0}+p_{0}} \end{array}$$

where we have used that $T_{0}^{\mu \nu }=\rho_{0}U^{\mu }U^{\nu }+p_{0}\Delta^{\mu \nu }$, $\Delta^{\mu \nu }=\eta^{\mu \nu }+U^{\mu }U^{\nu }$ and finally, using the Stefan–Boltzmann relation,

(61) $$\frac{\delta T}{T_{0}}=\frac{1}{4}\frac{\delta \rho }{\rho_{0}}$$

(62) $$\delta \beta_{\nu }=\frac{1}{T_{0}}\delta u_{\nu }-\frac{U_{\nu }}{T_{0}^{2}}\delta T=-\frac{\Delta_{\rho }^{\nu }\delta T^{\rho \sigma }U_{\sigma }}{T_{0}\left(\rho_{0}+p_{0}\right)}-\frac{U_{\nu }}{4T_{0}\rho_{0}}\delta T^{\rho \sigma }U_{\rho }U_{\sigma }$$

Putting it all together,

(63) $$\begin{array}{ccc} \delta I_{col} & = & \frac{1}{\tau }U_{\mu }p^{\mu }\left[\delta f-\delta f_{0}\right]\\ & = & -{\left(2\pi \right)}^{3}\frac{p^{0}}{\tau }f_{0}\left[J+\frac{3}{4\rho_{0}T_{0}}p^{\nu }\left[\Delta_{\nu \rho }+\frac{1}{3}U_{\nu }U_{\rho }\right]\int \;Dp^{\prime }\;p^{\prime \rho }U_{\sigma }p^{\prime \sigma }f_{0}J\right] \end{array}$$

where we have used the equation of state $p_{0}=\frac{\rho_{0}}{3}$.

In summary, if we assume the collision integral acquires a stochastic component

(64) $$I_{col}\to I_{col}+I\left(\left(t,x^{j}\right),p_{j}\right)$$

then, from the fluctuation–dissipation theorem (52),

(65) $$\begin{array}{ccc} & & \langle I\left(\left(t,x^{j}\right),p_{j}\right)I\left(\left(t^{\prime },y^{k}\right),q_{k}\right)\rangle =\frac{2}{\tau }\left(-u_{\mu }p^{\mu }\right)\left(-u_{\mu }q^{\mu }\right)f_{0}\left(p\right)\delta \left(x-y\right)\delta \left(t-t^{\prime }\right)\\ & & \left\{{\left(2\pi \right)}^{3}\delta \left(p-q\right)-\frac{3f_{0}\left(q\right)p^{\nu }q^{\rho }}{4\rho_{0}T_{0}}\left[\Delta_{\nu \rho }+\frac{1}{3}u_{\nu }u_{\rho }\right]\right\}. \end{array}$$

After taking moments, the hydrodynamic equations obtain noise terms [85,95]

(66) $$\begin{array}{ccc} I^{\mu } & = & \int \;Dp\;p^{\mu }I\\ I^{\mu \nu } & = & S_{\mu^{\prime }\nu^{\prime }}^{\mu \nu }\int \;Dp\;\frac{p^{\mu^{\prime }}p^{\nu^{\prime }}}{\left(-u_{\sigma }p^{\sigma }\right)}I\\ I^{\mu \nu \rho } & = & S_{\mu^{\prime }\nu^{\prime }\rho^{\prime }}^{\mu \nu \rho }\int \;Dp\;\frac{p^{\mu^{\prime }}p^{\nu^{\prime }}p^{\rho^{\prime }}}{{\left(-u_{\sigma }p^{\sigma }\right)}^{2}}I \end{array}$$

Now, we find

(67) $$\langle I^{\mu }\left(x\right)I^{\nu }\left(x^{\prime }\right)\rangle =\langle I^{\mu }\left(x\right)I^{\nu \rho }\left(x^{\prime }\right)\rangle =\langle I^{\mu }\left(x\right)I^{\nu \rho \sigma }\left(x^{\prime }\right)\rangle =0$$

so, we may simply take $I^{\mu }=0$. The noise does not feed energy, but entropy, into the system [25,26].

The remaining correlations are

(68) $$\begin{array}{ccc} \langle I^{\mu \nu }\left(x\right)I_{\lambda \tau }\left(x^{\prime }\right)\rangle & = & \frac{4}{15\tau }\tilde{\rho }S_{\lambda \tau }^{\mu \nu }\delta \left(x-x^{\prime }\right)\\ \langle I^{\mu \nu }\left(x\right)I_{\lambda \tau \omega }\left(x^{\prime }\right)\rangle & = & 0\\ \langle I^{\mu \nu \rho }\left(x\right)I_{\lambda \tau \omega }\left(x^{\prime }\right)\rangle & = & \frac{4}{35\tau }\tilde{\rho }S_{\lambda \tau \omega }^{\mu \nu \rho }\delta \left(x-x^{\prime }\right) \end{array}$$

where $\tilde{\rho }=12T^{5}/\pi^{2}$.

We observe that the noise in the hydrodynamic equations is additive; however, for a more realistic collision integral [96,97] there will be multiplicative noise too [98,99]. We aim to discuss this issue in future publications.

### 3.4. MSR Hydrodynamics

To set up the MSR action corresponding to a viscous relativistic fluid, we introduce Lagrange multipliers $Y_{\mu }$, $Y_{\mu \nu }$ and $Y_{\mu \nu \rho }$, the latter being transverse and traceless. Then, the action reads

(69) $$\begin{array}{ccc} S & = & \int \;d^{4}x\;\left\{-Y_{\mu ,\nu }T^{\mu \nu }-Y_{\mu \nu ,\rho }A^{\mu \nu \rho }-Y_{\mu \nu }\left[K^{\mu \nu \sigma \lambda }u_{\sigma ,\lambda }+I^{\mu \nu }\right]-Y_{\mu \nu \rho ,\sigma }A^{\mu \nu \rho \sigma }\right.\\ & - & \left.Y_{\mu \nu \rho }\left[2K^{\mu \nu \rho \sigma \lambda }u_{\sigma ,\lambda }+I^{\mu \nu \rho }\right]+2i\frac{\tilde{\rho }}{\tau }\left[\frac{1}{15}Y^{\mu \nu }Y_{\mu \nu }+\frac{1}{35}Y^{\mu \nu \rho }Y_{\mu \nu \rho }\right]\right\} \end{array}$$

So far, the treatment is fully nonlinear. However, we know from the Navier–Stokes equations that the most relevant nonlinear terms are those related to convective derivatives, over and above corrections to the viscous energy momentum tensor. To capture that kind of behavior, we shall linearize on $X^{\mu \nu }$ and $X^{\mu \nu \rho }$, while leaving $u^{\mu }$ arbitrary; then, we define

(70) $$u^{\mu }=\mu U^{\mu }+v^{\mu }$$

where $U^{\mu }=\delta_{0}^{\mu }$, $U^{\mu }v_{\mu }=0$ and $\mu =1+\frac{1}{2}v^{\mu }v_{\mu }+$ higher order. Then, $\Delta^{00}=v^{k}v_{k}$, $\Delta^{0k}=\mu v^{k}$, $\Delta^{jk}=\delta^{jk}+v^{j}v^{k}$. Observe that, similarly, $U_{\mu }X^{\mu \nu }=-v_{\mu }X^{\mu \nu }$ is a second-order quantity, while $U_{\mu }U_{\nu }X^{\mu \nu }=v_{\mu }v_{\nu }X^{\mu \nu }$ is of third order.

Given the complexity of the theory, we shall produce a demonstrative calculation retaining only some of the relevant Feynman graphs.

Our goal is to see how radiative corrections affect the tensor fluctuations. We cannot build the theory out of tensor modes alone, because tensor modes couple to each other through vector modes. The simplest nontrivial theory has two vector modes and two tensor modes, namely, the vector part of $v^{k}$ (therefore, we assume that $v_{,k}^{k}=0$), the vector and tensor parts of $X^{jk}$ (to single out, which we assume $X_{jk}=x_{j,k}+x_{k,j}+{\bar{x}}_{jk}$, with $x_{,j}^{j}={\bar{x}}_{,k}^{jk}=0$) and the tensor part of $X_{jkl}={\bar{x}}_{jk,l}^{\prime }+{\bar{x}}_{kl,j}^{\prime }+{\bar{x}}_{lj,k}^{\prime }$ where ${\bar{x}}^{\prime }{\;}_{,k}^{jk}=0$. To obtain equations for them, we perform a similar decomposition of the Lagrange multipliers, namely, $Y_{j}=y_{j}$, $Y_{jk}=y_{j,k}^{\prime }+y_{k,j}^{\prime }+{\bar{y}}_{jk}$ and $Y_{jkl}={\bar{y}}_{jk,l}^{\prime }+{\bar{y}}_{kl,j}^{\prime }+{\bar{y}}_{lj,k}^{\prime }$. Because of rotation invariance, there are no nontrivial correlations between vector and tensor variables. Finally, we shall keep only one interaction, namely, the coupling between $v^{k}$, ${\bar{x}}^{jk}$ and ${\bar{y}}^{jk}$, which comes from the convective term. Now, the action reads

(71) $$\begin{array}{ccc} S & = & \int \;d^{4}x\;\left\{y_{j}\left[\frac{4}{3}\rho {\dot{v}}^{j}+\frac{2}{15}\tilde{\rho }\;\Delta x^{j}\right]\right.\\ & - & 2y_{j}^{\prime }\Delta \left[\frac{2}{15}\tilde{\rho }\left[{\dot{x}}^{j}+\frac{1}{\tau }x^{j}\right]+\frac{4\rho }{15}v^{j}\right]\\ & + & {\bar{y}}_{jk}\left[\frac{2}{15}\tilde{\rho }\left[{\dot{\bar{x}}}^{jk}+\frac{1}{\tau }{\bar{x}}^{jk}\right]+\frac{2}{35}\tilde{\rho }\;\Delta {\bar{x}}^{{}^{\prime }jk}\right]\\ & - & {\bar{y}}_{jk}^{\prime }\frac{6}{35}\tilde{\rho }\Delta \left[{\dot{\bar{x}}}^{{}^{\prime }jk}+\frac{1}{\tau }{\bar{x}}^{{}^{\prime }jk}+\frac{1}{3}{\bar{x}}^{jk}\right]\\ & + & \left.2i\frac{\tilde{\rho }}{15\tau }\left[-2y_{j}^{\prime }\Delta y_{j}^{\prime }+{\bar{y}}_{jk}{\bar{y}}_{jk}\right]-2i\frac{\tilde{\rho }}{35\tau }y^{{}^{\prime }jk}\Delta y_{jk}^{\prime }+{\bar{y}}_{jk}\frac{2}{15}\tilde{\rho }v^{l}{\bar{x}}_{,l}^{jk}\right\} \end{array}$$

Integrating out the $x^{j}$ and ${\bar{x}}^{{}^{\prime }jk}$ fields, we obtain the constraints

(72) $$\begin{array}{ccc} \frac{2}{15}\tilde{\rho }\;\Delta y_{j}+\frac{4}{15}\tilde{\rho }\Delta \left[{\dot{y}}_{j}^{\prime }-\frac{1}{\tau }y_{j}^{\prime }\right] & = & 0\\ \frac{2}{35}\tilde{\rho }\;\Delta {\bar{y}}_{jk}+\frac{6}{35}\tilde{\rho }\Delta \left[{\dot{y}}_{jk}^{\prime }-\frac{1}{\tau }y_{jk}^{\prime }\right] & = & 0 \end{array}$$

We use these constraints to elliminate $y_{j}$ and ${\bar{y}}_{jk}$, whereby

(73) $$\begin{array}{ccc} S & = & \int \;d^{4}x\;\left\{\frac{8}{3}\rho y_{j}^{\prime }\left[\left(\frac{\partial }{\partial t}+\frac{1}{\tau }\right){\dot{v}}^{j}-\frac{1}{5}\Delta v^{j}\right]\right.\\ & + & \frac{2}{5}\tilde{\rho }y_{jk}^{\prime }\left[{\left(\frac{\partial }{\partial t}+\frac{1}{\tau }\right)}^{2}{\bar{x}}^{jk}-\frac{1}{7}\Delta {\bar{x}}^{jk}\right]\\ & + & 2i\frac{\tilde{\rho }}{15\tau }\left[-2y_{j}^{\prime }\Delta y_{j}^{\prime }+9\left[\frac{\partial }{\partial t}-\frac{1}{\tau }\right]y_{jk}^{\prime }\left[\frac{\partial }{\partial t}-\frac{1}{\tau }\right]y^{{}^{\prime }jk}-\frac{9}{7}y^{{}^{\prime }jk}\Delta y_{jk}^{\prime }\right]\\ & - & \left.\frac{2}{5}\tilde{\rho }\left[{\dot{y}}_{jk}^{\prime }-\frac{1}{\tau }y_{jk}^{\prime }\right]v^{l}{\bar{x}}_{,l}^{jk}\right\} \end{array}$$

## 4. Perturbative Evaluation of the 2PIEA

We shall now evaluate the corrections to the “classical” correlations derived from the action (73), to first order in the loop expansion.

We are building graphs with three kinds of internal lines, corresponding to the velocity symmetric correlation $\langle vv\rangle$, the tensor symmetric correlation $\langle \bar{x}\bar{x}\rangle$ and the tensor causal propagator $\langle \bar{x}y^{\prime }\rangle$; cubic vertices where the incoming lines are one of each kind; and external lines that may be of two types, $\bar{x}$ or $y^{\prime }$. We are interested in contributions to the self-energy, whereby one external line is of $y_{jk}^{\prime }$ type and the other of ${\bar{x}}^{jk}$, and corrections to the noise kernel, where both external lines are of the $y_{jk}^{\prime }$ kind. Therefore, we have the relationships

(74) $$\begin{array}{ccc} V & = & 2I_{VV}\\ V & = & I_{XY}+2I_{XX}+E_{X}\\ V & = & I_{XY}+E_{Y}\\ I_{VV}+I_{XX}+I_{XY}-V & = & L-1 \end{array}$$

where *V* is the number of vertices, *L* of loops, $I_{XX}$ the internal lines of the XX kind and $E_{X}$ the external lines of the *X* kind. The solution to this system reads

(75) $$\begin{array}{ccc} I_{VV} & = & L-1+\frac{1}{2}E_{X}+\frac{1}{2}E_{Y}\\ I_{XX} & = & -\frac{1}{2}E_{X}+\frac{1}{2}E_{Y}\\ I_{XY} & = & 2\left(L-1\right)+E_{X}\\ V & = & 2\left(L-1\right)+E_{X}+E_{Y} \end{array}$$

As τ→∞, the poles of the propagators move closer to the real axis, and this causes each loop integral to diverge linearly in τ in the free streaming limit. Therefore, we estimate the contribution of each loop integral as $\left(k\tau \right)k^{4}$. Including this factor, a given graph scales as

(76) $${\left(\frac{\tilde{\rho }}{\tau \rho^{2}k^{2}}\right)}^{I_{VV}}{\left(\frac{1}{\tau \tilde{\rho }k^{2}}\right)}^{I_{XX}}{\left(\frac{1}{\tilde{\rho }k^{2}}\right)}^{I_{XY}}{\left(\tilde{\rho }k^{2}\right)}^{V}{\left(\tau k^{5}\right)}^{L}$$

Rearranging, we obtain

(77) $$\left(\frac{\tau \rho^{2}k^{2}}{\tilde{\rho }}\right){\left(\frac{\tilde{\rho }}{\rho }\right)}^{E_{X}}{\left(\frac{\tilde{\rho }}{\tau \rho }\right)}^{E_{Y}}{\left(\frac{\tilde{\rho }k^{3}}{\rho^{2}}\right)}^{L}$$

Self energy graphs have $E_{X}=E_{Y}=1$; so,

(78) $$\Sigma \approx \tilde{\rho }k^{2}{\left(\frac{\tilde{\rho }k^{3}}{\rho^{2}}\right)}^{L}$$

A noise kernel graph has $E_{X}=0$, $E_{Y}=2$

(79) $$N\approx \left(\frac{\tilde{\rho }k^{2}}{\tau }\right){\left(\frac{\tilde{\rho }k^{3}}{\rho^{2}}\right)}^{L}$$

We see that the loop expansion is reliable for all momenta k≤T. In the following, we shall consider the first corrections to the self energy and the noise kernel as we approach the free streaming limit τ→∞.

In the opposite limit τ→0, the tensor modes disappear and the equations for the scalar and vector modes become constitutive relations appropriate to the Chapman–Enskog theory. Therefore, in that limit, we recover the analysis of refs. [100,101].

### 4.1. “Classical” Propagators

We define the Fourier transform in both time and space as

(80) $$\begin{array}{c} h^{j}\left(x\right)=\int \frac{d\omega }{2\pi }\;\frac{d^{3}k}{{\left(2\pi \right)}^{3}}e^{-i\left(\omega \;t-\vec{k}\cdot \vec{x}\right)}\;h^{j}\left(\omega ,\vec{k}\right) \end{array}$$

Due to isotropy and time-translation symmetry, the vector propagators read (where $h^{i}$ and $g^{j}$ are just two generic divergenceless vector fields)

(81) $$\begin{array}{c} \langle h^{i}\left(x\right)\;g^{j}\left(x^{\prime }\right)\rangle =\langle h^{i}g^{j}\rangle \left(t-t^{\prime },\vec{x}-{\vec{x}}^{\prime }\right) \end{array}$$

and, consequently,

(82) $$\begin{array}{c} \langle h^{i}\left(\omega ,\vec{k}\right)\;g^{j}\left(\omega^{\prime },{\vec{k}}^{\prime }\right)\rangle ={\left(2\pi \right)}^{4}\;\delta \left(\vec{k}+{\vec{k}}^{\prime }\right)\;\delta \left(\omega +\omega^{\prime }\right)\;\langle h^{i}g^{j}\rangle \left(\omega ,\vec{k}\right) \end{array}$$

where

(83) $$\begin{array}{c} \langle h^{i}g^{j}\rangle \left(\omega ,\vec{k}\right)=G_{hg}\left(\omega ,k\right)\;P^{ij}\left(\hat{k}\right) \end{array}$$

with

(84) $$\begin{array}{c} P^{ij}\left(\hat{k}\right)=\delta^{ij}-\frac{k^{i}k^{j}}{k^{2}}\; \end{array}$$

the vector spatial projector. In case of having tensor propagators, $P^{ij}$ must be replaced by the tensor spatial projector

(85) $$\begin{array}{c} P^{ijkl}=\left(P^{ik}P^{jl}+P^{il}P^{jk}-P^{ij}P^{kl}\right)/2\;. \end{array}$$

After Fourier transforming, the relevant propagators are

(86) $$\langle v^{j}y_{l}^{\prime }\rangle =\left(\frac{-3}{8\rho }\right)\frac{iP_{l}^{j}\left(\hat{k}\right)}{\left[\omega \left(\omega +\frac{i}{\tau }\right)-\frac{1}{5}k^{2}\right]}$$

(87) $$\langle {\bar{x}}^{jk}y_{lm}^{\prime }\rangle =\left(\frac{-5}{2\tilde{\rho }}\right)\frac{iP_{lm}^{jk}\left(\hat{k}\right)}{\left[{\left(\omega +\frac{i}{\tau }\right)}^{2}-\frac{1}{7}k^{2}\right]}$$

and the linear fluctuations are

(88) $$\begin{array}{ccc} \langle v^{j}v^{k}\rangle & = & \left(\frac{3\tilde{\rho }}{40\tau \rho^{2}}\right)\frac{k^{2}P^{jk}\left(\hat{k}\right)}{\left[{\left(\omega^{2}-\frac{1}{5}k^{2}\right)}^{2}+\frac{\omega^{2}}{\tau^{2}}\right]} \end{array}$$

(89) $$\begin{array}{ccc} \langle {\bar{x}}^{jk}{\bar{x}}^{lm}\rangle & = & \frac{15}{\tau \tilde{\rho }}\frac{\left(\omega^{2}+\frac{1}{\tau^{2}}+\frac{1}{7}k^{2}\right)P^{jklm}\left(\hat{k}\right)}{\left[{\left(\omega^{2}-\frac{k^{2}}{7}-\frac{1}{\tau^{2}}\right)}^{2}+\frac{4\omega^{2}}{\tau^{2}}\right]} \end{array}$$

At equal times, the fluctuation spectra are

(90) $$\begin{array}{ccc} {\langle v^{j}v^{k}\rangle }_{t=t^{\prime }} & = & \left(\frac{3\tilde{\rho }}{16\rho^{2}}\right)\;P^{jk}\left(\hat{k}\right) \end{array}$$

(91) $$\begin{array}{ccc} {\langle {\bar{x}}^{jk}{\bar{x}}^{lm}\rangle }_{t=t^{\prime }} & = & \left(\frac{15}{2\tilde{\rho }}\right)\;P^{jklm}\left(\hat{k}\right) \end{array}$$

which do not depend on τ as expected since, in equilibrium for equal times, the thermodynamic behavior is dominant over the dynamical effects.

### 4.2. Feynman Graphs

The lowest-order contribution to $\Gamma_{Q}$ is (cfr. Equation (27))

(92) $$\Gamma_{Q}=\frac{i}{2}{\left(\frac{2}{5}\tilde{\rho }\right)}^{2}\int d^{4}xd^{4}x^{\prime }\;{\langle \left[\left({\dot{y}}_{jk}^{\prime }-\frac{1}{\tau }y_{jk}^{\prime }\right)v^{l}{\bar{x}}_{,l}^{jk}\right]\left(x\right)\left[\left({\dot{y}}_{j^{\prime }k^{\prime }}^{\prime }-\frac{1}{\tau }y_{j^{\prime }k^{\prime }}^{\prime }\right)v^{l^{\prime }}{\bar{x}}_{,l^{\prime }}^{j^{\prime }k^{\prime }}\right]\left(x^{\prime }\right)\rangle }_{2PI}$$

namely,

(93) $$\begin{array}{ccc} & & \Gamma_{Q}=\frac{2i}{25}{\tilde{\rho }}^{2}\int d^{4}xd^{4}x^{\prime }\left\{\left(\frac{\partial }{\partial t}-\frac{1}{\tau }\right)\frac{\partial }{\partial x^{\prime l^{\prime }}}\langle y_{jk}^{\prime }\left(x\right){\bar{x}}^{j^{\prime }k^{\prime }}\left(x^{\prime }\right)\rangle \right.\\ & & \left(\frac{\partial }{\partial t^{\prime }}-\frac{1}{\tau }\right)\frac{\partial }{\partial x^{l}}\langle {\bar{x}}^{jk}\left(x\right)y_{j^{\prime }k^{\prime }}^{\prime }\left(x^{\prime }\right)\rangle \langle v^{l}\left(x\right)v^{l^{\prime }}\left(x^{\prime }\right)\rangle \\ & + & \left(\frac{\partial }{\partial t}-\frac{1}{\tau }\right)\left(\frac{\partial }{\partial t^{\prime }}-\frac{1}{\tau }\right)\langle y_{jk}^{\prime }\left(x\right)y_{j^{\prime }k^{\prime }}^{\prime }\left(x^{\prime }\right)\rangle \\ & & \left.\frac{\partial^{2}}{\partial x^{l}\partial x^{\prime l^{\prime }}}\langle {\bar{x}}^{jk}\left(x\right){\bar{x}}^{j^{\prime }k^{\prime }}\left(x^{\prime }\right)\rangle \langle v^{l}\left(x\right)v^{l^{\prime }}\left(x^{\prime }\right)\rangle \right\} \end{array}$$

#### 4.2.1. The Self Energy

The self energy is

(94) $$\Sigma_{j^{\prime }k^{\prime }}^{jk}=\frac{4i}{25}{\tilde{\rho }}^{2}P_{rs}^{jk}P_{j^{\prime }k^{\prime }}^{r^{\prime }s^{\prime }}\left(\frac{\partial }{\partial t}+\frac{1}{\tau }\right)\frac{\partial }{\partial x^{\prime l^{\prime }}}\frac{\partial }{\partial x^{l}}\left[\left(\frac{\partial }{\partial t^{\prime }}-\frac{1}{\tau }\right)\langle {\bar{x}}^{rs}\left(x\right)y_{r^{\prime }s^{\prime }}^{\prime }\left(x^{\prime }\right)\rangle \langle v^{l}\left(x\right)v^{l^{\prime }}\left(x^{\prime }\right)\rangle \right]$$

In Fourier space,

(95) $$\begin{array}{ccc} & & \Sigma_{j^{\prime }k^{\prime }}^{jk}\left(k\right)=\left(\frac{3i{\tilde{\rho }}^{2}}{100\tau \rho^{2}}\right)P_{\left(k\right)rs}^{jk}P_{\left(k\right)j^{\prime }k^{\prime }}^{r^{\prime }s^{\prime }}\left(-ik^{0}+\frac{1}{\tau }\right)k_{l}k_{l^{\prime }}\\ & & \int \frac{d\omega d^{3}p}{{\left(2\pi \right)}^{4}}\frac{P_{\left(p\right)r^{\prime }s^{\prime }}^{rs}\left(\omega +\frac{i}{\tau }\right)}{\left[{\left(\omega +\frac{i}{\tau }\right)}^{2}-\frac{1}{7}p^{2}\right]}\frac{{\left(k-p\right)}^{2}P_{\left(k-p\right)}^{ll^{\prime }}}{\left[{\left({\left(\omega -k^{0}\right)}^{2}-\frac{1}{5}{\left(k-p\right)}^{2}\right)}^{2}+\frac{{\left(\omega -k^{0}\right)}^{2}}{\tau^{2}}\right]} \end{array}$$

By symmetry, $\Sigma_{j^{\prime }k^{\prime }}^{jk}=\Sigma P_{\left(k\right)j^{\prime }k^{\prime }}^{jk}$; then,

(96) $$\begin{array}{ccc} & & \Sigma =\frac{1}{2}\Sigma_{ij}^{ij}=\left(\frac{3{\tilde{\rho }}^{2}}{200\tau \rho^{2}}\right)\left(k^{0}+\frac{i}{\tau }\right)\\ & & \int \frac{d\omega d^{3}p}{{\left(2\pi \right)}^{4}}\frac{P_{\left(k\right)rs}^{r^{\prime }s^{\prime }}P_{\left(p\right)r^{\prime }s^{\prime }}^{rs}\left(\omega +\frac{i}{\tau }\right)}{\left[{\left(\omega +\frac{i}{\tau }\right)}^{2}-\frac{1}{7}p^{2}\right]}\frac{{\left(k-p\right)}^{2}P_{\left(k-p\right)}^{ll^{\prime }}k_{l}k_{l^{\prime }}}{\left[{\left({\left(\omega -k^{0}\right)}^{2}-\frac{1}{5}{\left(k-p\right)}^{2}\right)}^{2}+\frac{{\left(\omega -k^{0}\right)}^{2}}{\tau^{2}}\right]} \end{array}$$

We may take $k^{i}=\delta_{3}^{i}k$ and then $p^{i}=\left(p_{⊥}^{a},p^{3}\right)$, a=1,2, whereby

(97) $$P_{\left(k-p\right)}^{ll^{\prime }}k_{l}k_{l^{\prime }}=\frac{k^{2}p_{⊥}^{2}}{{\left(k-p\right)}^{2}}$$

We also find

(98) $$P_{\left(k\right)r^{\prime }}^{r}P_{\left(p\right)s}^{r^{\prime }}=P_{\left(k\right)s}^{r}-\frac{p_{⊥}^{r}p_{s}}{p^{2}}$$

(99) $$P_{\left(k\right)rs}^{r^{\prime }s^{\prime }}P_{\left(p\right)r^{\prime }s^{\prime }}^{rs}=2-2\frac{p_{⊥}^{2}}{p^{2}}+\frac{1}{4}{\left(\frac{p_{⊥}^{2}}{p^{2}}\right)}^{2}$$

We can assume approximate isotropy and obtain

(100) $$P_{\left(k\right)rs}^{r^{\prime }s^{\prime }}P_{\left(p\right)r^{\prime }s^{\prime }}^{rs}\approx \frac{4}{5}$$

so, now,

(101) $$\Sigma =\left(\frac{3{\tilde{\rho }}^{2}}{250\tau \rho^{2}}\right)\left(k^{0}+\frac{i}{\tau }\right)k^{2}I$$

where

(102) $$I=\int \frac{d\omega d^{3}p}{{\left(2\pi \right)}^{4}}\frac{p_{⊥}^{2}\left(\omega +\frac{i}{\tau }\right)}{\left[{\left(\omega +\frac{i}{\tau }\right)}^{2}-\frac{1}{7}p^{2}\right]\left[{\left({\left(\omega -k^{0}\right)}^{2}-\frac{1}{5}{\left(k-p\right)}^{2}\right)}^{2}+\frac{{\left(\omega -k^{0}\right)}^{2}}{\tau^{2}}\right]}$$

We give details of the evaluation of the integral (102) in Appendix A. From the results there and the previous analysis (78), we find in the free streaming limit

(103) $$\Sigma =\frac{2i\tilde{\rho }}{5}{\left(k^{0}+\frac{i}{\tau }\right)}^{2}\left(\frac{\tilde{\rho }k^{3}}{\rho^{2}}\right)\sigma^{2}\left[\frac{k^{0}}{k}\right]$$

We will not need the precise form of the σ function in what follows, see Appendix A.

#### 4.2.2. The Noise Kernel

The noise kernel is

(104) $$\begin{array}{ccc} & & N_{j^{\prime }k^{\prime }}^{jk}\left(k\right)=\left(\frac{9{\tilde{\rho }}^{2}}{100\tau^{2}\rho^{2}}\right)P_{\left(k\right)rs}^{jk}P_{\left(k\right)r^{\prime }s^{\prime }}^{j^{\prime }k^{\prime }}\left({\left(k^{0}\right)}^{2}+\frac{1}{\tau^{2}}\right)k_{l}k_{l^{\prime }}\\ & & \int \frac{d\omega d^{3}p}{{\left(2\pi \right)}^{4}}\frac{\left(\omega^{2}+\frac{1}{\tau^{2}}+\frac{1}{7}p^{2}\right)P_{p}^{rsr^{\prime }s^{\prime }}}{\left[{\left(\omega^{2}-\frac{p^{2}}{7}-\frac{1}{\tau^{2}}\right)}^{2}+\frac{4\omega^{2}}{\tau^{2}}\right]}\frac{{\left(k-p\right)}^{2}P_{\left(k-p\right)}^{ll^{\prime }}}{\left[{\left({\left(\omega -k^{0}\right)}^{2}-\frac{1}{5}{\left(k-p\right)}^{2}\right)}^{2}+\frac{{\left(\omega -k^{0}\right)}^{2}}{\tau^{2}}\right]} \end{array}$$

We assume $N^{jkj^{\prime }k^{\prime }}=NP^{jkj^{\prime }k^{\prime }}$. Proceeding with the self energy, we find

(105) $$N=\left(\frac{9{\tilde{\rho }}^{2}}{250\tau^{2}\rho^{2}}\right)\left({\left(k^{0}\right)}^{2}+\frac{1}{\tau^{2}}\right)k^{2}I_{N}$$

where

(106) $$I_{N}=\int \frac{d\omega d^{3}p}{{\left(2\pi \right)}^{4}}\frac{\left(\omega^{2}+\frac{1}{\tau^{2}}+\frac{1}{7}p^{2}\right)}{\left[{\left(\omega^{2}-\frac{p^{2}}{7}-\frac{1}{\tau^{2}}\right)}^{2}+\frac{4\omega^{2}}{\tau^{2}}\right]}\frac{p_{⊥}^{2}}{\left[{\left({\left(\omega -k^{0}\right)}^{2}-\frac{1}{5}{\left(k-p\right)}^{2}\right)}^{2}+\frac{{\left(\omega -k^{0}\right)}^{2}}{\tau^{2}}\right]}$$

In the free streaming limit, we find

(107) $$N=\frac{12\tilde{\rho }}{5\tau }\left({\left(k^{0}\right)}^{2}+\frac{1}{\tau^{2}}\right)\left(\frac{\tilde{\rho }k^{3}}{\rho^{2}}\right)N\left[\frac{k^{0}}{k}\right]$$

see Appendix A and (79).

### 4.3. The Spectrum

The results so far may be summarized by saying that the self energy takes the form (103) while the correction to the noise kernel is (107). Therefore, the corrected symmetric propagator reads

(108) $$\langle {\bar{x}}^{jk}{\bar{x}}_{jk}\rangle =\frac{15}{\tau \tilde{\rho }}\frac{\left[\left({\left(k^{0}\right)}^{2}+\frac{1}{\tau^{2}}\right)\left[1+\left(\frac{\tilde{\rho }k^{3}}{\rho^{2}}\right)N\left[\frac{k^{0}}{k}\right]\right]+c_{T}^{2}k^{2}\right]}{{\left|{\left(k^{0}+\frac{i}{\tau }\right)}^{2}\left(1+\left(\frac{\tilde{\rho }k^{3}}{\rho^{2}}\right)\sigma^{2}\left[\frac{k^{0}}{k}\right]\right)-c_{T}^{2}k^{2}\right|}^{2}}$$

where $c_{T}^{2}=1/7$. We may speculate about the spectrum in a case where loop corrections would be dominant. In that case, we would obtain

(109) $$\langle {\bar{x}}^{jk}{\bar{x}}_{jk}\rangle =\frac{15\rho^{2}}{\tau {\tilde{\rho }}^{2}k^{3}}\frac{N\left(\frac{k^{0}}{k}\right)}{\left(k^{02}+\frac{1}{\tau^{2}}\right){\left|\sigma \left(\frac{k^{0}}{k}\right)\right|}^{2}}$$

To compute the equal time correlation, we must integrate over $k^{0}$, which in the free streaming limit adds a further factor of 1/k, and then at equal times

(110) $$\langle {\bar{x}}^{jk}{\bar{x}}_{jk}\rangle \propto \frac{\rho^{2}}{\tau {\tilde{\rho }}^{2}k^{4}}$$

Remarkably, power law spectra such as this are associated to entropy cascades, with a scale-invariant spectrum $k^{-3}$ corresponding to fully developed relativistic turbulence [26].

## 5. Final Remarks

When a nonlinear system is coupled to a random force, mode–mode coupling affects both the inertia of the system and the effective force felt by it. This shows up in such effects as long time tails.

The MSR approach is an efficient tool to incorporate these effects in a consistent way, and takes full advantage of methods developed to treat similar problems in quantum field theory.

In this paper, we have demonstrated the MSR approach by applying it to the calculation of the dressed thermal fluctuations of the non-hydrodynamic tensor mode of a relativistic viscous fluid.

The existence of such modes is a generic prediction of kinetic theory. Those modes play a leading role in the interaction between fluids and gravitational waves both in cosmological and astrophysical settings.

The dressing by loop corrections changes a flat spectrum for long wavelengths to a power law one at short wavelengths.

We believe these techniques will play an important role in the further analysis of phenomena involving relativistic viscous fluids and electromagnetic and gravitational fields, and look forward to report on further progress soon. 

## Data Availability

Not applicable.

## Appendix A. One Loop Feynman Graphs

In this appendix we give further details about the evaluation of the one-loop contributions to the self energy (102) and the noise kernel (106).

### Appendix A.1. Self Energy

We write Equation (102) as

(A1) $$I=\int \frac{d\omega d^{3}p}{{\left(2\pi \right)}^{4}}\;p_{⊥}^{2}\left(\omega +\frac{i}{\tau }\right){\langle xy\rangle }_{ret}{\langle vv\rangle }_{1}$$

where

(A2) $$\begin{array}{ccc} {\langle xy\rangle }_{ret} & = & \frac{1}{\left[{\left(\omega +\frac{i}{\tau }\right)}^{2}-\frac{1}{7}p^{2}\right]}\\ {\langle vv\rangle }_{1} & = & \frac{1}{\left[{\left({\left(\omega -k^{0}\right)}^{2}-\frac{1}{5}{\left(k-p\right)}^{2}\right)}^{2}+\frac{{\left(\omega -k^{0}\right)}^{2}}{\tau^{2}}\right]} \end{array}$$

*I* has units of *k*. We factorize

(A3) $${\langle vv\rangle }_{1}=\frac{-i\tau }{2\left(\omega -k^{0}\right)}\left[{\langle vv\rangle }_{ret}-{\langle vv\rangle }_{adv}\right]$$

where

(A4) $$\begin{array}{ccc} {\langle vv\rangle }_{ret} & = & \frac{1}{\left[{\left(\omega -k^{0}-\frac{i}{2\tau }\right)}^{2}-\frac{1}{5}{\left(k-p\right)}^{2}+\frac{1}{4\tau^{2}}\right]}\\ {\langle vv\rangle }_{adv} & = & \frac{1}{\left[{\left(\omega -k^{0}+\frac{i}{2\tau }\right)}^{2}-\frac{1}{5}{\left(k-p\right)}^{2}+\frac{1}{4\tau^{2}}\right]} \end{array}$$

Now

(A5) $$\int \frac{d\omega d^{3}p}{{\left(2\pi \right)}^{4}}\;p_{⊥}^{2}\left(\omega +\frac{i}{\tau }\right)\frac{-i\tau }{\left(\omega -k^{0}\right)}{\langle xy\rangle }_{ret}{\langle vv\rangle }_{adv}=0$$

so

(A6) $$I=\frac{1}{2}\int \frac{d\omega d^{3}p}{{\left(2\pi \right)}^{4}}\;p_{⊥}^{2}\left(\omega +\frac{i}{\tau }\right)\frac{-i\tau }{\left(\omega -k^{0}\right)}{\langle xy\rangle }_{ret}{\langle vv\rangle }_{ret}$$

It is convenient to write

(A7) $$\frac{\left(\omega +\frac{i}{\tau }\right)}{\left(\omega -k^{0}\right)}=1+\left(k^{0}+\frac{i}{\tau }\right)\frac{\left(\omega +k^{0}\right)}{\left(\omega^{2}-k^{02}\right)}$$

so correspondingly

(A8) $$I=\frac{1}{2}\left(-i\tau \right)\left[I_{1}+\left(k^{0}+\frac{i}{\tau }\right)I_{2}\right]$$

where

(A9) $$I_{1}=\int \frac{d\omega d^{3}p}{{\left(2\pi \right)}^{4}}\;p_{⊥}^{2}{\langle xy\rangle }_{ret}{\langle vv\rangle }_{ret}$$

(A10) $$I_{2}=\int \frac{d\omega d^{3}p}{{\left(2\pi \right)}^{4}}\;p_{⊥}^{2}\frac{\left(\omega +k^{0}\right)}{\left(\omega^{2}-k^{02}\right)}{\langle xy\rangle }_{ret}{\langle vv\rangle }_{ret}$$

$I_{1}$ has units of $k^{2}$, $I_{2}$ has units of *k*.

### Appendix A.2. Noise Kernel

The noise kernel (106) may be analyzed in a similar way.

(A11) $$N=\left(\frac{9{\tilde{\rho }}^{2}}{250\tau^{2}\rho^{2}}\right)\left({\left(k^{0}\right)}^{2}+\frac{1}{\tau^{2}}\right)k^{2}I_{N}$$

where $I_{N}$ is dimensionless

(A12) $$I_{N}=\int \frac{d\omega d^{3}p}{{\left(2\pi \right)}^{4}}\left(\omega^{2}+\frac{1}{\tau^{2}}+\frac{1}{7}p^{2}\right)p_{⊥}^{2}{\langle xx\rangle }_{1}{\langle vv\rangle }_{1}$$

${\langle vv\rangle }_{1}$ as in Equation (A2), and

(A13) $${\langle xx\rangle }_{1}=\frac{1}{\left[{\left(\omega^{2}-\frac{p^{2}}{7}-\frac{1}{\tau^{2}}\right)}^{2}+\frac{4\omega^{2}}{\tau^{2}}\right]}$$

${\langle vv\rangle }_{1}$ may be handled as in Equation (A3), and

(A14) $${\langle xx\rangle }_{1}=\frac{i\tau }{4\omega }\left\{{\langle xy\rangle }_{ret}-{\langle xy\rangle }_{adv}\right\}$$

(A15) $$\begin{array}{ccc} {\langle xy\rangle }_{ret} & = & \frac{1}{\left[{\left(\omega +\frac{i}{\tau }\right)}^{2}-c_{T}^{2}p^{2}\right]}\\ {\langle xy\rangle }_{adv} & = & \frac{1}{\left[{\left(\omega -\frac{i}{\tau }\right)}^{2}-c_{T}^{2}p^{2}\right]} \end{array}$$

It follows that

(A16) IN=τ24Re∫dωd3p2π4ω2+1τ2+17p2p⊥2ωω−k0vvretxyret

The integral is equal to $I_{1}+I_{3}$, where $I_{1}$ is given by (A9), and $I_{3}$, which scales as $k^{2}$,

(A17) $$I_{3}=\int \frac{d\omega d^{3}p}{{\left(2\pi \right)}^{4}}\frac{\left(\omega k^{0}+\frac{1}{\tau^{2}}+\frac{1}{7}p^{2}\right)p_{⊥}^{2}}{\left(\omega^{2}-\omega k^{0}\right)}{\langle vv\rangle }_{ret}{\langle xy\rangle }_{ret}$$

### Appendix A.3. Computing the Integrals

We now elaborate on the computation of $I_{1}$. Introducing Feynman parameters [102]

(A18) $$I_{1}=\int_{0}^{1}dx\int \frac{d\omega d^{3}p}{{\left(2\pi \right)}^{4}}\;\frac{p_{⊥}^{2}}{D_{\Sigma 1}^{2}}$$

(A19) $$D_{\Sigma 1}={\left(\omega +\frac{i}{\tau }\left(1-\frac{3}{2}x\right)-k^{0}x\right)}^{2}+\Omega^{2}+\frac{3ik^{0}}{\tau }x\left(1-x\right)$$

(A20) $$\Omega^{2}=M^{2}-\left[xc_{V}^{2}+\left(1-x\right)c_{T}^{2}\right]\left({\left(p_{z}-\delta p_{z}\right)}^{2}+p_{⊥}^{2}\right)$$

$c_{T}=1/\sqrt{7}$, $c_{V}=1/\sqrt{5}$,

(A21) $$\delta p_{z}=\frac{xc_{V}^{2}k}{\left[xc_{V}^{2}+\left(1-x\right)c_{T}^{2}\right]}$$

(A22) $$M^{2}=x\left(1-x\right)\left[k^{02}-\frac{c_{V}^{2}c_{T}^{2}}{\left[xc_{V}^{2}+\left(1-x\right)c_{T}^{2}\right]}k^{2}\right]-\frac{1}{\tau^{2}}2x\left(1-\frac{9}{8}x\right)$$

We shift $\omega \to \omega +xk^{0}$ and $p_{z}\to p_{z}+\delta p_{z}$.

(A23) $$I_{1}=\int_{0}^{1}dx\int \frac{d\omega d^{3}p}{{\left(2\pi \right)}^{4}}\;\frac{p_{⊥}^{2}}{{\left[{\left(\omega +\frac{i}{\tau }\left(1-\frac{3}{2}x\right)\right)}^{2}+M^{2}-C^{2}\left[x\right]p^{2}+\frac{3ik^{0}}{\tau }x\left(1-x\right)\right]}^{2}}$$

where

(A24) $$C\left[x\right]=\sqrt{xc_{V}^{2}+\left(1-x\right)c_{T}^{2}}$$

We rescale *p* and go to polar coordinates

(A25) $$I_{1}=\frac{4}{3}\int_{0}^{1}\frac{dx}{C^{5}\left[x\right]}\int \frac{d\omega dp}{{\left(2\pi \right)}^{3}}\;\frac{p^{4}}{{\left[{\left(\omega +\frac{i}{\tau }\left(1-\frac{3}{2}x\right)\right)}^{2}+M^{2}-p^{2}+\frac{3ik^{0}}{\tau }x\left(1-x\right)\right]}^{2}}$$

Then the integral has (double) poles at

(A26) $$\omega_{\pm }=-\frac{i}{\tau }\left(1-\frac{3}{2}x\right)\pm i\omega_{0}$$

(A27) $$\omega_{0}=\sqrt{M^{2}-p^{2}+\frac{3ik^{0}}{\tau }x\left(1-x\right)}$$

If both poles lie on the same half plane, then the integral vanishes.

If x<2/3, we close the countour from above, catching the pole at $\omega =\omega_{+}$

(A28) $$I_{1}^{<}=\frac{1}{3}\int_{0}^{2/3}\frac{dx}{C^{5}\left[x\right]}\int \frac{dp\;p^{4}}{{\left(2\pi \right)}^{2}}\;\frac{1}{\omega_{0}^{3}}$$

The integral is dominated by the value $p_{0}$ of *p* such that Imω+ is barely above zero. We approximate

(A29) $$\int dp\;\frac{p^{4}}{\omega_{0}^{\alpha }}=\frac{p_{0}^{3}}{\left(\alpha -2\right)\omega_{0}^{\alpha -2}}$$

We then have

(A30) $$\int dp\;\frac{p^{4}}{\omega_{0}^{3}}\approx \frac{p_{0}^{3}}{\omega_{0}}$$

Write

(A31) $$\omega_{0}\left[p_{0}\right]=\frac{1}{\tau }\left(1-\frac{3}{2}x\right)+i\xi$$

Taking the square of both terms and equating the imaginary parts

(A32) $$\frac{2\xi }{\tau }\left(1-\frac{3}{2}x\right)=\frac{3k^{0}}{\tau }x\left(1-x\right)$$

Observe that ξ is independent of τ

(A33) $$\xi =\frac{3k^{0}}{2}\frac{x\left(1-x\right)}{\left(1-\frac{3}{2}x\right)}$$

and so

(A34) $$p_{0}^{2}=M^{2}+\xi^{2}-\frac{1}{\tau^{2}}{\left(1-\frac{3}{2}x\right)}^{2}$$

In the τ→∞ limit we get

(A35) $$\int \frac{dp\;p^{4}}{{\left(2\pi \right)}^{2}}\;\frac{1}{\omega_{0}^{3}}\approx -i\frac{{\left[M^{2}+\xi^{2}\right]}^{3/2}}{{\left(2\pi \right)}^{2}\xi }$$

Which is finite when x→0 but diverges when x→2/3. This latter divergence is canceled by a divergence with opposite sign coming from the integral with x>2/3.

We see that the leading term (A35) is imaginary, so the contribution to the noise kernel comes from the next to leading order in the expansion

(A36) $$\frac{1}{\omega_{0}}\left[p_{0}\right]=\frac{-i}{\xi }+\frac{1}{\tau \xi^{2}}\left(1-\frac{3}{2}x\right)+\ldots$$

$I_{2}$ (A10) and $I_{3}$ (A17) are computed in the same way. The presence of extra factors in the denominators is handled by adding one more Feynman parameter.
